# Dengue in Travelers: Kinetics of Viremia and NS1 Antigenemia and Their Associations with Clinical Parameters

**DOI:** 10.1371/journal.pone.0065900

**Published:** 2013-06-03

**Authors:** Elina O. Erra, Essi M. Korhonen, Liina Voutilainen, Eili Huhtamo, Olli Vapalahti, Anu Kantele

**Affiliations:** 1 Division of Infectious Diseases, Department of Medicine, Helsinki University Central Hospital, Helsinki, Finland; 2 Department of Bacteriology and Immunology, Haartman Institute, Faculty of Medicine, University of Helsinki, Helsinki, Finland; 3 Department of Virology, Haartman Institute, Faculty of Medicine, University of Helsinki, Helsinki, Finland; 4 Finnish Forest Research Institute, Vantaa Research Unit, Vantaa, Finland; Duke-National University of Singapore Graduate Medical School, Singapore

## Abstract

Despite the increasing numbers of travel-acquired dengue, few studies have assessed virologic markers of the disease in non-endemic populations. We examined the kinetics of diagnostic markers and their associations with clinical parameters in 93 patients with travel-acquired dengue fever. Kinetics analyses suggested a longer average duration for viremia (9 days, CI95%: 8–10) and non-structural protein 1 (NS1) antigenemia (15 days, CI95%: 12–20) than reported in endemic populations. While none of the tests sufficed alone, the best diagnostic coverage was achieved by combining antibody detection with RNA or NS1 testing. Studied by regression models, early relative levels of viremia and NS1 antigenemia proved to be significantly associated with several clinical parameters: high viremia predicted greater likelihood and increased length of hospitalization, the degree of NS1 antigenemia correlated positively with hematocrit and liver transaminases, and both viremia and NS1 antigenemia levels negatively with platelet counts in follow-up. Levels of viremia and NS1 antigenemia may serve as predictors of the clinical manifestations in travel-acquired dengue.

## Introduction

Dengue is the most common mosquito-borne viral disease worldwide, causing 50–100 million infections annually [1]. It is endemic in most tropical and subtropical parts of the world, especially in urban areas, many of which are popular travel destinations. Accordingly, ongoing dengue epidemics have manifested themselves as an increasing number of infections not only in endemic populations, but also among travelers returning from the dengue endemic areas [2], [3].

The laboratory diagnostics of dengue virus (DENV) infections is currently based on virus isolation, and detection of DENV RNA, non-structural protein 1 (NS1), and DENV-specific antibodies [4], [5]. Few laboratories can provide the full range, yet none of these assays covers the entire disease period [4], [5]. For a rational choice and timing of the tests, the kinetics of the various diagnostic markers needs to be understood. For efficient diagnostics, it appears that two or more methods should be combined.

The clinical outcome of DENV infections ranges in severity from asymptomatic or non-specific febrile illness to classical dengue fever and to severe dengue characterized by one or more of the following: plasma leakage, severe bleeding, and severe organ impairment [5]. Severe dengue infections should be treated in dedicated high-dependency units, where a mortality of less than 1% can be achieved [3]. In travelers, dengue is rarely a life-threatening disease; yet, severe forms of disease are also seen [6], [7].

Epidemiologic studies have identified several risk factors for severe disease, including previous exposure to heterologous DENV serotype, infection with certain viral strains and serotypes, young age, female sex, and certain gene variants of the host [8]–[12]. A high degree of serum viremia and NS1 antigenemia have been associated with a more severe disease outcome in endemic populations [13]–[17]. Differing results have also been reported [18], [19], though, and studies in travel-acquired dengue have been lacking.

Recently, the importance of early diagnosis and risk prediction of clinical outcome have been emphasized [3]. In addition to endemic areas, these should also be studied among travelers who may differ from endemic populations in a number of essential respects, such as lack of previous immunity against heterotypic serotypes, dissimilarity in pre-existing immunity against other flaviviruses (e.g. Japanese encephalitis, tick-borne encephalitis, yellow fever), variety of infecting DENV sero- and genotypes, as well as age and genetic background.

The present study investigated the clinical and diagnostic data of 93 Finnish travelers with acute dengue infections, aiming at (1) describing the kinetics of DENV viremia, NS1 antigenemia, and DENV-specific antibodies, (2) assessing their use in diagnostics as combinations, and (3) examining the potential correlation between diagnostic markers (viremia and NS1 antigenemia) and clinical parameters in travel-acquired dengue.

## Methods

### Ethics statement

As a retrospective registry study based solely on patient files and routine, archived blood samples, instead of patient consent and an ethics approval, the study required research clearances from the institutes and the Ministry of Social Affairs and Health. Accordingly, the study was approved by the research boards of the Department of Internal Medicine of Helsinki University Central Hospital, Helsinki University Hospital laboratory (HUSLAB), and the Ministry of Social Affairs and Health.

### Study design

In Finland, laboratory diagnostics of dengue has been based on serological testing performed in a single laboratory for all the patients throughout the country (HUSLAB, Helsinki University Central Hospital). For this study, we retrospectively recorded data on all the DENV IgM-positive patients in Finland 1999–2008 (a total of 154 cases). From these patients, all samples taken within 21 days since illness onset were collected, including the early IgM-negative sera. Four patients were excluded due to suspicion of false positive IgM. This group consisted of one published case of Japanese encephalitis [20], one laboratory-confirmed tick-borne encephalitis (strong positive anti-TBE IgM in serum and cerebrospinal fluid along with a characteristic clinical picture, and only weakly positive serum anti-dengue IgM), and 2 patients who did not show diagnostic seroconversion in serial samples. After additionally excluding those with insufficient clinical information (57 patients), the data comprised a total of 139 samples from 93 patients (1–3 samples per patient). The first samples from a subgroup of patients (72/93) were evaluated previously [21]; these early-phase sera were included in the present study along with convalescent samples for analyses of the kinetics of virologic markers, and with additional clinical data for studying the associations between virologic markers and clinical parameters.

The clinical and laboratory data were analyzed by two kinds of statistical approaches: (1) those exploring the kinetics of diagnostic markers either with a single-test approach or as combinations of two tests, and (2) those examining the associations between virologic markers and clinical parameters. For analyses of the kinetics, we included all 139 serum samples taken within 21 days since the onset of symptoms (93 patients, 1–3 samples each). For the association analyses, we included a subgroup of 89 patients with data on the following potential confounding factors: age, sex, day of illness, presence of co-infections and chronic diseases, which were controlled in the statistical analyses.

### Collection of clinical data

The following clinical information was retrieved retrospectively from medical records: demographic characteristics (age, gender, country of birth), chronic diseases (long-lasting condition that can be controlled but not cured, such as diabetes mellitus), previous vaccinations against other flaviviruses, travel destination, symptoms, results of routine laboratory investigations, presence of co-infections (additional diagnosis of an acute infectious disease other than dengue), possible hospitalization and its length. The date of the onset of symptoms was designated as day 1. Laboratory parameters were classified as being normal, decreased, or elevated according to current reference values. The severity of the cases was assessed according to the WHO dengue classification criteria valid at the time of data collection [22].

### Virological and serological assays

The anti-DENV IgM tests were performed with a commercial enzyme immunoassay (EIA) (Focus Technologies) following manufacturer's instructions. The anti-DENV IgGs were determined by an in-house immunofluorescence assay as previously described [23]. The tests were carried out as dilution series of 1∶10 to 1∶640, employing acetone-fixed DENV-3 infected Vero E6 cells as the antigen.

RNA extraction, the detection of RNA by real-time reverse transcription polymerase chain reaction (RT-PCR) (tested in 132 samples), and of the NS1 antigen by EIA (tested in 135 samples) were performed as previously described [21]. The serum aliquots were stored at −70°C and −20°C for RNA and NS1 detection, respectively. The RNA extractions were carried out from 100 µl of serum specimens by the QIAamp Viral RNA Mini Kit (Qiagen) according to manufacturer's instructions. For the DENV NS1 antigen detection we used the commercial Platelia Dengue NS1 Ag EIA assay (Bio-Rad) adhering to manufacturer's instructions. Some of the NS1 Ag tests (69/135) and one-step real-time RT-PCRs (69/132) were carried out separately prior to this study [21]. For the 63 sera tested for RNA for this study only, the Stratagene thermocycler was used instead of the ABI Prism 7700 Sequence Detection System. The comparability and reproducibility of the results obtained with the two pieces of equipment was ascertained by means of appropriate internal controls. The infecting DENV serotype was determined by virus isolation, DENV-typing RT-PCR [24] and consequent sequencing [21], [25], or by direct sequencing of RT-PCR products [24], [26] obtained directly from serum samples.

The default setting for Finnish travelers is a primary infection, as the vast majority are – apart from a vaccinated minority – flavivirus naïve. As high levels of IgG with low or even absent IgM levels during the early acute phase have been associated with secondary infections [5], for the purpose of this study, cases with positive RT-PCR result, IgG titers ≥80 (IFA), and a negative IgM (IgM-EIA) test in the acute phase were considered as possible secondary cases.

### Statistical analyses

All statistical analyses were performed with R software [27]. The kinetics of serum DENV RNA, NS1 antigen, and DENV-specific antibodies were studied using generalized additive mixed models (GAMM) [28]. In all models, a smoothing spline of the number of days since illness onset was employed as the explanatory variable. Two kinds of univariate models were formed: (1) those with Gaussian error distributions and an identity link function, where continuous values of diagnostic markers (inverse cycle threshold [ct] -value, NS1 ratio, IgM index, and log_10_-transformed IgG titre) were used as dependent variables, and (2) those with binomial error distributions and a logit link function with binary outcomes (negative/positive) of the four diagnostic tests as dependent variables. Similarly, we studied the kinetics of different combinations of two diagnostic tests over the course of illness, using GAMM with binomial error distribution and a logit link function, the classes of the dependent variable being negative in both tests/positive in either of the tests.

Associations between virologic markers (serum DENV RNA and NS1 antigen) and clinical parameters (routine laboratory test results, symptoms during follow-up, likelihood and length of hospitalization) were studied by regression models. The full model had the clinical parameter as the dependent variable, and potential confounding factors (age, sex, day of illness, presence of co-infections and chronic diseases) as explanatory variables. Each of the virologic markers (serum DENV RNA and NS1 antigen) were included in the full model in turn, and a model set constituting 95% of Akaike weights of all models nested within the full model was then averaged [29] using MuMIN package [30] in R software. Gaussian error distributions, an identity link function, and continuous diagnostic explanatory variables (the inverse ct-value from RT-PCR [cut-off ct-value – sample ct-value], NS1 ratio from antigen EIA) were applied to models with continuous values of routine laboratory findings as dependent variables. A Poisson error distribution adjusted for overdispersion, a log link function, and continuous diagnostic variables were used in models studying the duration of hospitalization. For models examining binary dependent variables (likelihood of abnormal laboratory findings/occurrence of different symptoms/hospitalization), we employed binomial error distributions, a logit link function, and binary diagnostic outcomes (serum DENV RNA/NS1 positivity). Explanatory variables were considered statistically significant (on 0.05 level), if the 95% confidence intervals of their coefficients excluded zero.

## Results

### Patient characteristics

Table 1 and Table 2 summarize the background characteristics and clinical data of the study population. The majority of the patients were Finnish adults with no underlying chronic diseases. Most of the dengue cases were acquired in Asia (72%).

**Table 1:** **Background characteristics of the 93 travelers with dengue.**

		%	N
Gender			
Male		56	52/93
Female		44	41/93
Ethnic origin			
Finnish		88	82/93
Other		12	11/93
Chronic diseasesa			
Generally healthy		79	72/91
Chronic diseases		21	19/91
History of previous flavivirus vaccinationb			
Japanese encephalitis vaccine		11	10/92
Yellow fever vaccine		20	18/92
Tick-borne encephalitis vaccine		1	1/92
Any of the above		22	20/92
Geographic region visitedb			
South-East Asia		50	46/92
South Central Asia		21	19/92
Central America and Caribbean		14	13/92
Sub-Saharan Africa		8	7/92
South America		7	6/92
Southwest Asia		1	1/92

The median age of patients was 37 years (interquartile range: 28 to 45 years).

a Data missing for two patients.

b Data missing for one patient.

**Table 2:** **Clinical characteristics of the 93 travelers with dengue.**

		%	N
Clinical symptoms			
Fever		93	86/92
Rash		71	65/92
Headache		64	59/92
Myalgia		55	51/92
Fatigue		51	47/92
Other gastrointestinal symptoms		46	42/92
Nausea		45	41/92
Arthralgia		34	31/92
Respiratory symptoms		27	25/92
Hemorrhagic manifestations		24	22/92
Vomiting		24	22/92
Retro-orbital pain		17	16/92
Pruritus		9	8/90
Shock		0	0/92
Routine laboratory findings in follow-up^a^			
Anaemia (<134/117 g/L)		9	5/56
Elevated Hb (>167/155 g/L)		13	11/87
Low Hcr (<39%/35%)		9	5/56
Elevated Hcr (>50%/46%)		8	7/87
Leukopenia (<3.4×10^9^/L)		67	59/88
Thrombocytopenia (<150×10^9^/L)		78	68/87
Elevated AST (>45/35 U/L)		78	49/63
Elevated ALT (>70/45 U/L)		61	51/83
Elevated creatinine (>100/90 µmol/L)		25	10/40
Patients positive for the various diagnostic tests^b^			
PCR		74%	67/90
NS1		79%	72/91
IgM^c^		100%	93/93
IgG		98%	91/93
Serotype identified			
DENV-1		26	24/93
DENV-2		8	7/93
DENV-3		24	22/93
DENV-4		3	3/93
Not known		40	37/93
Co-infections^d^			
At least one co-infection		19	17^e^/91
Gastrointestinal infection		9	8/91
Respiratory tract infection		4	4/91
Urinary tract infection		2	2/91
Other bacterial disease		2	2/91
Malaria		2	2/91
Other parasitic disease		1	1/91
Hospitalization		79	73^f^/92

a Reference values for males/females in parentheses.

b At least once during the 21 days since illness onset. The timing of serum sampling was not standardized.

c Selection criterion for entering the study.

d Diagnosed according to current practice.

e Of these patients, 9/17 (53%) were positive for PCR or NS1 at some point. The patients negative for PCR and NS1 provided samples on illness days 6–21.

f Duration of hospitalization: median 4 days, interquartile range 3 to 6 days.

Abbreviations: ALT, alanine transaminase; AST, aspartate transaminase; DENV, dengue virus; Hb, hemoglobin; Hcr, hematocrit.

All patients were classified as having an uncomplicated dengue infection. Most of the cases were categorized as primary infections. Only three patients were considered to have a probable secondary infection: in the first sample they had a considerable anti-DENV IgG level concomitantly with a negative anti-DENV IgM, yet their samples were positive for DENV-RNA. No other cases with a considerable early IgG and low or non-existent IgM were detected. None of the suspected secondary cases had received flaviviral vaccines in the past.

The majority (79%) were admitted to hospital, the mean length of hospitalization being 4.5 (SD 2.5) days. Their treatment was symptomatic, and some patients were also given antimicrobials, either because they were initially suspected to have a bacterial infection or because a simultaneous co-infection (19% [17/91] of the cases) was diagnosed. The most common co-infection was bacterial gastroenteritis.

All four DENV serotypes were detected in this patient population (Table 2). DENV-1 (24/56) and DENV-3 (22/56) were the predominant serotypes, accounting for 82% (46/56) of cases with identified serotype.

### Kinetics of diagnostic markers

**Figure 1:**
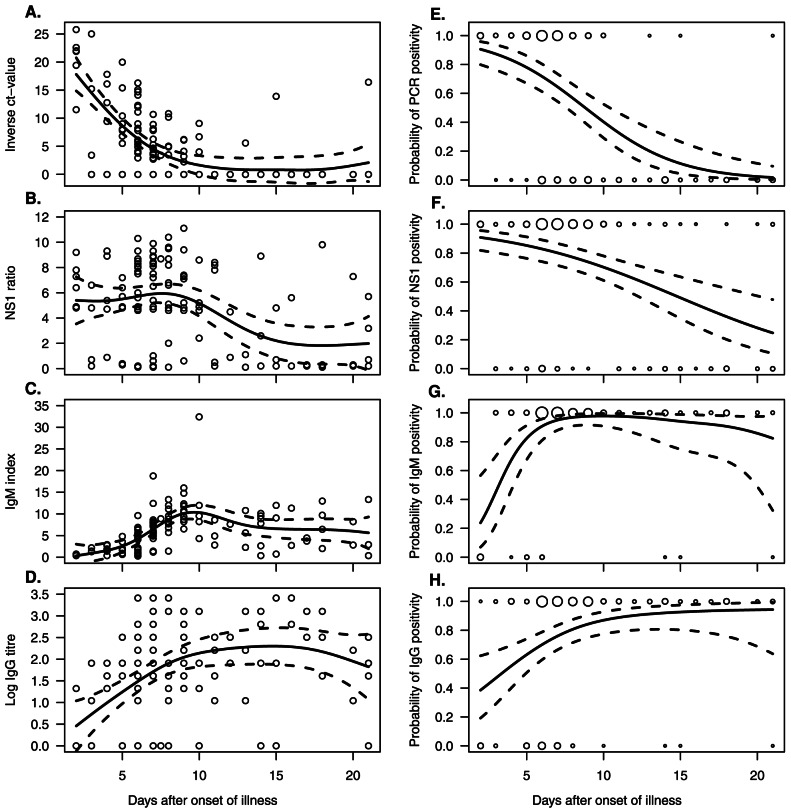
Kinetics of diagnostic markers. Relative amounts (A–D) and the probability of a positive result (E–H) of DENV-RNA, NS1 protein, and DENV specific IgM and IgG antibodies in the serum samples of 93 patients with acute dengue fever. Solid lines indicate predicted means (A–D), and probabilities (E–H) from generalized additive mixed models (GAMM), and dashed lines their 95% confidence intervals. In A–D, the circles serve to illustrate individual observations; in E–H, the circles show the positive/negative test results at each given time point, the size of the circle being proportional to the number of observations.

Figure 1 shows the kinetics of plasma viremia, NS1 antigenemia, and DENV-specific IgM and IgG antibodies in the 93 patients studied. DENV RNA and NS1 antigen were detected already on the 2^nd^ day of illness (the earliest serum samples), with a probability of 91% (95% CI: 82–96% for PCR positivity and 80–96% for NS1 positivity) (Figure 1E, 1F). On the 7th day of illness, 65% (95% CI: 54–74%) of the sera were still positive for DENV RNA, and 80% (95% CI: 71–87%) were positive for NS1. Patients were likely to remain PCR positive up to illness day 9 (95% CI: day 8–10), whereas NS1 positivity would last longer, until illness day 15 (95% CI: day 12–20). On day 21, none of the sera proved positive for DENV RNA, while 20% were still positive for NS1 (Figure 1E, 1F).

During the first 12 days of illness, all serum samples from patients with DENV-1 infection (n = 35), but only 69% (n = 32) of those from patients with DENV-3 infection were NS1 positive (p = 0.0003, Fisher's exact test). In the 13 samples taken after day 12, this difference was no longer seen. The kinetics of viremia showed no serotype-dependent differences (data not shown).

**Figure 2:**
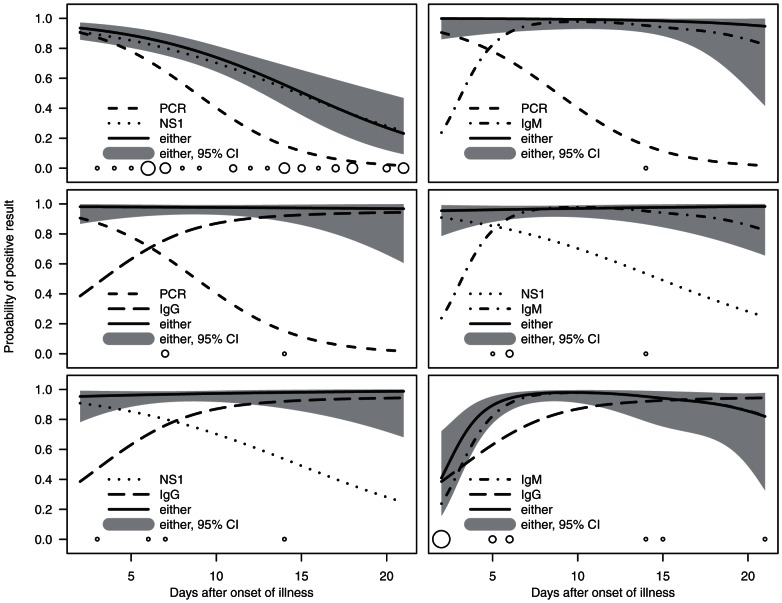
Kinetics of diagnostic combinations. The solid lines and shaded areas provide the predicted probabilities and their 95% confidence intervals for at least one of the two diagnostic tests showing a positive result on a given day of illness. Non-solid lines indicate the predicted probabilities of positive result for each test alone. The circles illustrate time points of samples negative in both tests, the size of the circle being proportional to the number of observations.

Figure 2 shows the sensitivities for the combinations of two diagnostic tests over the first three weeks of illness. When RNA detection was combined with IgM detection, only 1% (1/120) of the serum samples remained negative (the negative sample taken on illness day 14). The combination of NS1 and IgM detection remained negative in 3% (4/120) of the samples. Although not primarily intended for identifying acute infection, the combinations with IgG detection were also studied. When either RNA or NS1 tests were used together with IgG detection, 2% (3/129) or 3% (4/130) of the samples were negative, respectively. When combining IgM and IgG detection, the results proved negative for 10% (12/124) of the samples; when using RNA and NS1 detection together, 25% (32/127) proved negative.

### Associations between virologic markers and clinical parameters

Associations between virologic markers (serum viremia/NS1 antigenemia) and clinical parameters were studied with regression models in a subgroup of 89 patients who provided data on the following potential confounding factors controlled in the analyses (age, sex, day of illness, presence of co-infections and chronic diseases). All associations reported below were statistically significant on the 0.05 level when confounding factors were controlled for. Table S1, and Figures S1 and S2 show the associations between virologic markers and clinical parameters. Tables S2 and S3 provide the averaged coefficients of all explanatory variables and their 95% CIs for all models studied.

#### Associations between viremia and clinical parameters

While controlling for potential confounding factors, e.g. day of illness, relative levels of DENV RNA in the first serum sample correlated negatively with leukocyte and platelet nadirs in follow-up (Figure S1, Table S3). In addition, overall PCR positivity at the time of presentation predicted a higher probability of leukopenia, thrombocytopenia, and elevated alanine and aspartate transaminases (ALT, AST) during the illness (Tables S1 and S2).

No associations were found between initial DENV RNA positivity and the various symptoms during the illness (Table S2).

Patients found DENV RNA positive at the time of admission proved more likely to be hospitalized than RNA negative patients (Tables S1 and S2). Furthermore, a high relative amount of serum DENV RNA at the time of presenting to hospital increased both the probability and duration of hospitalization (Figure S2, Table S3).

#### Associations between NS1 antigenemia and clinical parameters

Relative levels of NS1 antigen at the time of admission correlated negatively with leukocyte and platelet nadirs, and positively with maximum levels of hemoglobin, hematocrit and liver transaminases (AST, ALT) in follow-up (Figure S1, Table S3). Overall NS1 positivity at the time of hospital admission was associated with a higher probability of leukopenia, thrombocytopenia, elevated ALT, and AST (Tables S1 and S2). The odds ratios for developing leukopenia, thrombocytopenia, or elevated ALT, were at least twice as high for RNA positive as for NS1 positive patients (Table S1).

NS1 positivity at the time of admission was found to be associated with fatigue and abdominal pain/diarrhea over the course of illness (Table S2).

No association was found between early NS1 positivity or relative amount of serum NS1 and probability/duration of hospitalization (Tables S1, S2, S3, Figure S2).

## Discussion

The need for better diagnostics of dengue and prediction of disease outcome is becoming increasingly urgent [3]. On account of differences in patient characteristics, separate data on endemic populations and travelers from non-endemic regions are essential. Our previous study suggested that the kinetics of DENV viremia and NS1 antigenemia may differ in traveler populations. This study further explores the kinetics of diagnostic markers, as well as their use as combinations, and correlations with clinical parameters in travel-acquired dengue.

### Kinetics of viremia

The current data suggest a longer span of serum viremia in travel-acquired dengue than that reported in endemic populations [4], [5], [16], [17]. Viral RNA was detectable on average until illness day 9; in endemic settings an average duration of 5 to 7 days has been reported [4], [5], [16], [17]. This finding might be related to the high proportion of primary infections among travelers, the clearance of viremia being slower in primary than secondary infections [16], [17].

### Kinetics of NS1 antigenemia

NS1 antigen was already detectable in the earliest serum samples, consistent with previous studies in endemic areas [31]–[33]. However, as in viremia, the NS1 positivity lasted longer than that reported in endemic populations. This might be due to slower clearance of the antigen in primary infections, or a higher sensitivity of the assay in the absence of pre-existing anti-NS1 antibodies. Compared to an average duration of 5 to 7 days recorded in endemic settings [5], [19], [31], [32], NS1 positivity lasted on average until illness day 15, the span of NS1 antigen detection being also significantly longer than that of serum viremia.

Over the first 12 days of illness, NS1 positivity was found more often in patients infected with DENV-1 than DENV-3, consistent with some previous studies [19], [34]. The NS1 EIA test is supposed to detect NS1 antigens of all serotypes equally [31]; a better sensitivity for serotype 1 has been suggested, however [17]. Altogether, the long detection span of NS1, availability of the test, and high specificity in comparison to antibody testing [31], [35] make the assessment of NS1 a useful tool for diagnosing traveler's dengue.

### Diagnostic coverage

When scrutinizing the first 3 weeks after onset, none of the diagnostic methods could alone serve as a sufficient diagnostic tool for the whole time period, as expected. Combining RNA or NS1 detection and antibody testing resulted in a high overall sensitivity, which accords with results from previous studies [19], [21], [33], [36], [37]. In settings where RNA detection is not readily available, the combination of IgM and NS1 appears to be a feasible approach. The advantages of NS1 over RNA detection are low cost and ease of testing. Moreover, the long detection span of NS1 antigen enhances diagnostic specificity in the later phase of the disease.

### Virologic markers and clinical picture

Dengue in travelers is considered mostly a mild disease [6], yet severe forms of the disease are occasionally seen [7]. None of our cases were classified as dengue hemorrhagic fever or dengue shock syndrome according to the WHO criteria valid at the time of data collection. A significant proportion of the study patients were hospitalized, however; the decision was taken on the basis of the clinicians' evaluation of the patients' general condition. In Finland it is customary to hospitalize febrile travelers whenever there is any reason to believe that their clinical status may get worse, regardless of whether a diagnosis has been established or not. In most cases dengue was not diagnosed until after the patient was admitted to hospital.

Despite the fact that the dengue cases were predominantly mild, regression analyses revealed several associations between the relative levels of virologic markers (DENV RNA and NS1) and the various clinical parameters studied. The initial levels of viremia predicted both likelihood and duration of hospitalization. Notably, the clinicians were unaware of their patients' viremia (or NS1 antigenemia) status at the time of illness, and the number of days from the onset of illness was controlled in the statistical analyses; if not, both of these could have had a confounding effect on the associations observed.

The initial levels of NS1 antigenemia were not associated with hospitalization, but correlated positively with some central laboratory parameters, such as hematocrit and liver transaminases. Notably, both serum viremia and NS1 antigenemia levels correlated negatively with platelet counts over the course of illness.

Studies conducted in endemic settings have related high serum viremia and NS1 antigenemia with severe dengue [13]–[17]; this relation has not been confirmed in some other studies, though [18], [19]. To our knowledge, correlations between virologic and clinical markers have not previously been assessed in travelers. To study these associations, a prospective study design with standardized data collection would have been superior to the retrospective approach. Due to the relatively low incidence of dengue among Finnish travelers, a prospective study would have been difficult to conduct in the current setting, though. One of the limitations of the retrospective research approach was the lack of comprehensive data on pre-existing flaviviral immunity. However, other known potential confounding factors (day of illness, and age, sex, and presence of co-infections and chronic diseases) were controlled in the analyses exploring the correlations between virologic and clinical markers. Despite infections being predominantly mild in this study, the observed correlations with clinical parameters (e.g. hematocrit and thrombocytopenia) suggest that viremia and NS1 antigenemia may serve as predictors of the clinical manifestations in travel-acquired dengue.

### Conclusions

This study suggests a longer average duration of serum viremia and NS1 antigenemia in travel-acquired dengue than that observed in endemic areas, emphasizing the importance of carrying out individual studies among different patient populations. Our results highlight the importance of combining diagnostic tests, such as NS1 and IgM detection, to provide better diagnostic coverage. The early appearance and long detection span of NS1, easy performance and rapidity of the test, as well as high specificity for dengue viruses as compared to antibody testing make the assessment of NS1 a feasible tool for diagnosing traveler's dengue. Both viremia and NS1 antigenemia showed correlation with several clinical parameters, suggesting a potential role in predicting disease outcome in traveler patients.

## Supporting Information

Figure S1 **Correlations between virologic markers and laboratory parameters.** Associations between relative amounts of DENV RNA and NS1 antigen in the first serum sample, and the minimum/maximum values of hemoglobin, leukocytes, platelets, ALT, and AST during follow-up. Lines indicate predictions from averaged linear models (thick line  =  statistically significant, thin line  =  non-significant association), and open symbols the observed data. In hemoglobin plots, solid line and open dots indicate the predictions and data for women, dashed lines and open squares those for men.
(EPS)

Figure S2 **Correlations between virologic markers and hospitalization.** Associations between relative amounts of serum DENV RNA/NS1 antigen at the time of presenting to hospital and probability and length of hospitalization. The circles indicate the degree of serum viremia/NS1 antigenemia for individual patients at the time of presenting to hospital and the choice of treatment place (out-patient/hospitalized)/length of hospitalization. The size of the circles is presented in proportion to the number of observations.
(EPS)

Table S1 **Odds ratios (OR) and predicted occurrence of abnormal laboratory values during follow-up depending on initial RNA or NS1 positivity.**
(DOCX)

Table S2 **Parameter estimates and their 95% confidence intervals from averaged model sets exploring the connection between the initial DENV RNA/NS1 antigen positivity and the probabilities of abnormal clinical parameters, hospitalization, and different symptoms during follow-up.**
(DOCX)

Table S3 **Parameter estimates and their 95% confidence intervals from averaged model sets exploring the connection between initial amounts of serum DENV RNA/NS1 antigen and clinical parameters in follow-up.**
(DOCX)
