# Supplementary material for: Dengue in Travelers: Kinetics of Viremia and NS1 Antigenemia and Their Associations with Clinical Parameters
Source: PLoS One. 2013 Jun 3;8(6):e65900. doi: 10.1371/journal.pone.0065900 (PMC3670861; doi:10.1371/journal.pone.0065900)
Supplement: Table S1 — Odds ratios (OR) and predicted occurrence of abnormal laboratory values during follow-up depending on initial RNA or NS1 positivity. (DOCX) [file pone.0065900.s003.docx]

| **Supplementary Table 1.** Odds ratios (OR) and predicted occurrence of abnormal laboratory values during follow-up depending on initial RNA or NS1 positivity. | | | | | | |
| --- | --- | --- | --- | --- | --- | --- |
|  | **OR^a^ (95% CI)^b^** | | **Predicted occurrence (%)^a^ (95% CI)^b^** | | | |
|  | RNA +/- | NS1 +/- | RNA+ | RNA- | NS1+ | NS1- |
| elevated Hb | 1.3  (0.3-5.8) | 1.0  (0-∞) | 11.5  (2.8-36.6) | 9.0  (1.5-38.7) | 9.1  (0.0-100.0) | 0.0  (0.0-100.0) |
| elevated Hcr | 1.1  (0-∞) | 1.1  (0-∞) | 9.1  (0.0 -100.0) | 0.0  (0.0-100.0) | 9.8  (0.0-100.0) | 0.0  (0.0-100.0) |
| leukopenia | **10.0**  **(2.9-34.1)** | **4.3**  **(1.1-17.1)** | **83.5**  **(59.6-94.5)** | **33.6**  **(9.4-71.1)** | **75.6**  **(43.9-92.5)** | **41.8**  **(18.7-69.2)** |
| thrombocytopenia | **22.6**  **(5.1-101.5)** | **10.9**  **(2.6-45.2)** | **97.8**  **(91.1-99.5)** | **66.7**  **(17.1-95.1)** | **98.2**  **(93.0-99.6)** | **83.3**  **(42.6-97.1)** |
| elevated ALT | **15.6**  **(2.9-84.8)** | **4.4**  **(1.1-17.6)** | **70.5**  **(30.6-92.8)** | **13.2**  **(2.4-48.0)** | **68.3**  **(34.6-89.6)** | **32.9**  **(10.2-67.8)** |
| elevated AST | **12.7**  **(1.8-90.0)** | **13.2**  **(2.2-77.5)** | **98.3**  **(89.2-99.8)** | **82.2**  **(20.9-98.8)** | **98.9**  **(93.7-99.8)** | **86.9**  **(34.5-98.8)** |
| elevated creatinine | 1.0  (0.2-4.8) | 0.9  (0.2-4.2) | 14.8  (3.4-46.5) | 15.3  (1.3-71.1) | 15.3  (3.8-45.5) | 16.7  (1.8-68.5) |
| hospitalization | **6.4**  **(1.9-22.2)** | 1.1  (0.5-2.1) | **85.5**  **(63.1-95.3)** | **47.8**  **(19.6-77.6)** | 80.3  (67.7-88.7) | 79.2  (52.7-92.9) |

Significant differences are written in bold.

^a^Odds ratios and proportions are calculated for an average-aged female with no co-infections and normal health on day 2 after illness onset

^b^95% CI = 95% confidence intervals of averaged coefficients

Abbreviations: ALT, alanine transaminase; AST aspartate transaminase; Hb, hemoglobin; Hcr, hematocrit; NS1, non-structural protein 1
