# Supplementary material for: Dengue in Travelers: Kinetics of Viremia and NS1 Antigenemia and Their Associations with Clinical Parameters
Source: PLoS One. 2013 Jun 3;8(6):e65900. doi: 10.1371/journal.pone.0065900 (PMC3670861; doi:10.1371/journal.pone.0065900)
Supplement: Table S2 — Parameter estimates and their 95% confidence intervals from averaged model sets exploring the connection between the initial DENV RNA/NS1 antigen positivity and the probabilities of abnormal clinical parameters, hospitalization, and different symptoms during follow-up. (DOCX) [file pone.0065900.s004.docx]

| **Supplementary Table 2.**  **Parameter estimates with their 95% confidence intervals from averaged model sets exploring the connection between the initial DENV RNA / NS1 antigen positivity and the probabilities of abnormal clinical parameters, hospitalization, and different symptoms during follow-up.** | | | | | | | | |
| --- | --- | --- | --- | --- | --- | --- | --- | --- |
|  | | Coefficients (95% CIs) | | | | | | |
|  | | **Elevated ALT^a^** | **Elevated AST^a^** | **Leukopenia^a^** | **Thrombo-**  **cytopenia^a^** | **Elevated**  **Hcr^a^** | **Elevated**  **Hb^a^** | **Elevated creatinine^a^** |
| **DENV RNA MODEL PARAMETERS** | | | |  |  |  |  |  |
|  | N patients analyzed | 78 | 59 | 83 | 82 | 82 | 82 | 36 |
|  | Intercept | -1.88  (-3.69, -0.08) | 1.53  (-1.33, 4.39) | -0.68  (-2.26, 0.90) | 0.70  (-1.58, 2.97) | -12.50  (-∞, ∞) | -2.31  (-4.16, -0.46) | -1.71  (-4.32, 0.90) |
|  | Presence of  co-infection | 1.63  (-0.48, 3.73) | 0.21  (-1.05, 1.48) | -0.14  (-0.99, 0.71) | 0.05  (-0.84, 0.94) | -7.31  (-∞, ∞) | -11.60  (-∞, ∞) | 0.13  (-0.98, 1.23) |
|  | *DENV RNA detected in first sample* | **2.75**  **(1.06, 4.44)** | **2.54**  **(0.58, 4.50)** | **2.30**  **(1.07, 3.53)** | **3.12**  **(1.63, 4.62)** | 10.20  (-∞, ∞) | 0.27  (-1.23, 1.76) | -0.04  (-1.65, 1.57) |
|  | Male gender | -0.06  (-0.63, 0.52) | **-3.28**  **(-5.90, -0.66)** | 0.04  (-0.51, 0.60) | 0.05  (-0.73, 0.84) | 0.13  (-0.88, 1.13) | 0.21  (-0.83, 1.26) | 1.62  (-0.52, 3.76) |
|  | Presence of chronic disease | 0.33  (-0.86, 1.52) | 0.47  (-1.24, 2.18) | 0.08  (-0.65, 0.80) | -0.15  (-1.22, 0.92) | 0.10  (-0.90, 1.10) | 0.04  (-0.78, 0.86) | -0.05  (-1.06, 0.96) |
|  | Days from onset to first RNA sample | 0.01  (-0.07, 0.08) | 0.04  (-0.14, 0.22) | -0.03  (-0.15, 0.09) | -0.16  (-0.37, 0.06) | -0.03  (-0.22, 0.17) | 0.00  (-0.10, 0.10) | -0.03  (-0.21, 0.15) |
|  | Age (mean-centered) | -0.01  (-0.05, 0.03) | 0.01  (-0.05, 0.07) | 0.00  (-0.03, 0.02) | 0.00  (-0.03, 0.03) | 0.02  (-0.04, 0.08) | 0.03  (-0.04, 0.09) | -0.01  (-0.05, 0.04) |
| **DENV NS1 MODEL PARAMETERS** | | | |  |  |  |  |  |
|  | N patients analyzed | 77 | 59 | 82 | 81 | 81 | 81 | 35 |
|  | Intercept | -0.71  (-2.17, 0.74) | 1.89  (-0.64, 4.43) | 0.45  (-0.51, 2.42) | 1.61  (-0.30, 3.51) | -11.80  (-∞, ∞) | -15.40  (-∞, ∞) | -1.61  (-4.01, 0.78) |
|  | Presence of co-infection | 0.61  (-0.93, 2.15) | 0.25  (-1.10, 1.61) | -0.33  (-1.47, 0.81) | -0.01  (-0.85, 0.84) | -7.75  (-∞, ∞) | -9.86  (-∞, ∞) | 0.33  (-1.11, 1.77) |
|  | *DENV NS1 detected in first sample* | **14.48**  **(0.08, 2.87)** | **2.58**  **(0.81, 4.35)** | **1.46**  **(0.08, 2.84)** | **2.39**  **(0.97, 3.81)** | 9.58  (-∞, ∞) | 13.10  (-∞, ∞) | -0.10  (-1.63, 1.43) |
|  | Male gender | -0.03  (-0.55, 0.49) | **-3.32**  **(-5.96, -0.68)** | 0.01  (-0.52, 0.54) | -0.01  (-0.74, 0.71) | 0.14  (-0.91, 1.19) | 0.65  (-1.09, 2.40) | 0.86  (-1.15, 2.88) |
|  | Presence of chronic disease | 0.11  (-0.62, 0.84) | 0.15  (-0.94, 1.23) | 0.02  (-0.61, 0.65) | -0.22  (-1.40, 0.96) | 0.05  (-0.87, 0.97) | 0.04  (-0.77, 0.86) | -0.05  (-1.03, 0.94) |
|  | Days from onset to first NS1 sample | -0.01  (-0.08, 0.06) | 0.01  (-0.10, 0.11) | -0.09  (-0.26, 0.08) | **-0.24**  **(-0.43, -0.06)** | -0.04  (-0.26, 0.18) | 0.00  (-0.12, 0.12) | -0.01  (-0.15, 0.13) |
|  | Age (mean-centered) | -0.01  (-0.04, 0.02) | 0.02  (-0.05, 0.10) | 0.00  (-0.02, 0.02) | 0.01  (-0.04, 0.06) | 0.02  (-0.04, 0.08) | 0.01  (-0.04, 0.06) | 0.00  (-0.04, 0.04) |
|  |  |  |  |  |  |  |  |  |
|  |  | Coefficients (95% CIs) | | | | | | |
|  |  | **Hospitali-**  **zation^a^** | **Days hospitalized^b^** | **Fever^a^** | **Headache^a^** | **Myalgia^a^** | **Fatigue^a^** | **Diarrhea/ abdominal pain^a^** |
| **DENV RNA MODEL PARAMETERS** | | | |  |  |  |  |  |
|  | N patients analyzed | 86 | 83 | 86 | 86 | 86 | 86 | 86 |
|  | Intercept | -0.09  (-1.41, 1.24) | 1.04  (0.42, 1.65) | 2.30  (0.27, 4.33) | 0.92  (-0.36, 2.19) | -0.11  (-1.57, 1.36) | -0.97  (-3.18, 1.25) | -0.12  (-1.46, 1.22) |
|  | Presence of co-infection | 0.17  (-0.80, 1.14) | 0.10  (-0.28, 0.47) | 0.17  (-1.26, 1.60) | -1.02  (-2.51, 0.47) | -0.20  (-1.07, 0.67) | -0.07  (-0.72, 0.59) | -0.04  (-0.62, 0.53) |
|  | *DENV RNA detected in first sample* | **1.86**  **(0.62, 3.10)** | 0.29  (-0.28, 0.86) | 0.68  (-1.24, 2.61) | 0.01  (-0.57, 0.60) | 0.36  (-0.71, 1.44) | 0.90  (-0.63, 2.42) | 0.22  (-0.67, 1.12) |
|  | Male gender | -0.07  (-0.74, 0.59) | -0.05  (-0.31, 0.20) | 0.11  (-0.85, 1.08) | -0.67  (-1.88, 0.54) | -0.26  (-1.08, 0.57) | -0.25  (-1.09, 0.60) | -0.45  (-1.45, 0.56) |
|  | Presence of chronic disease | 1.36  (-1.14, 3.86) | 0.20  (-0.27, 0.66) | 0.12  (-1.12, 1.36) | 0.77  (-0.79, 2.33) | 0.04  (-0.51, 0.59) | 0.15  (-0.65, 0.94) | -0.50  (-1.76, 0.75) |
|  | Days from onset to first RNA sample | 0.00  (-0.07, 0.08) | 0.00  (-0.03, 0.03) | -0.02  (-0.15, 0.10) | 0.02  (-0.08, 0.11) | 0.02  (-0.08, 0.12) | 0.06  (-0.09, 0.21) | 0.02  (-0.07, 0.10) |
|  | Age (mean-centered) | 0.00  (-0.02, 0.02) | 0.00  (-0.01, 0.01) | -0.02  (-0.09, 0.04) | -0.02  (-0.06, 0.03) | 0.00  (-0.02, 0.02) | 0.02  (-0.02, 0.07) | 0.00  (-0.02, 0.02) |
| **DENV NS1 MODEL PARAMETERS** | | | |  |  |  |  |  |
|  | N patients analyzed | 86 | 83 | 86 | 86 | 86 | 86 | 86 |
|  | Intercept | 1.34  (0.11, 2.57) | 1.30  (0.88, 1.73) | 2.97  (0.89, 5.04) | 0.66  (-0.59, 1.92) | -0.82  (-2.71, 1.08) | -2.19  (-4.54, 0.17) | -1.73  (-3.92, 0.46) |
|  | Presence of co-infection | -0.01  (-0.67, 0.64) | 0.06  (-0.24, 0.36) | 0.07  (-1.12, 1.27) | -0.97  (-2.45, 0.51) | -0.18  (-1.04, 0.69) | -0.03  (-0.66, 0.61) | 0.01  (-0.62, 0.64) |
|  | *DENV NS1 detected in first sample* | 0.06  (-0.60, 0.72) | 0.01  (-0.22, 0.24) | -0.12  (-1.40, 1.16) | 0.10  (-0.59, 0.78) | 1.22  (-0.22, 2.66) | **2.00**  **(0.47, 3.52)** | **1.73**  **(0.23, 3.23)** |
|  | Male gender | -0.15  (-0.92, 0.62) | -0.09  (-0.40, 0.23) | 0.07  (-0.82, 0.96) | -0.44  (-1.53, 0.65) | -0.27  (-1.14, 0.59) | -0.24  (-1.10, 0.62) | -0.33  (-1.26, 0.61) |
|  | Presence of chronic disease | 1.42  (-0.99, 3.82) | 0.16  (-0.28, 0.60) | 0.14  (-1.13, 1.42) | 0.91  (-0.70, 2.51) | -0.05  (-0.61, 0.52) | 0.07  (-0.59, 0.74) | -0.49  (-1.73, 0.75) |
|  | Days from onset to first NS1 sample | -0.02  (-0.12, 0.08) | -0.01  (-0.04, 0.03) | -0.04  (-0.19, 0.11) | 0.02  (-0.07, 0.11) | 0.04  (-0.08, 0.17) | 0.10  (-0.08, 0.27) | 0.06  (-0.09, 0.22) |
|  | Age (mean-centered) | 0.00  (-0.02, 0.03) | 0.00  (-0.01, 0.01) | -0.02  (-0.08, 0.04) | -0.02  (-0.06, 0.03) | 0.00  (-0.02, 0.02) | 0.02  (-0.02, 0.06) | 0.00  (-0.02, 0.02) |
|  |  | Coefficients (95% CIs) | | | | | | |
|  |  | **Nausea^a^** | **Arthralgia^a^** | **Respiratory**  **symptoms^a^** | **Hemorrhagic**  **symptoms^a^** | **Vomiting^a^** | **Retroorbital pain^a^** | **Pruritus^a^** |
| **DENV RNA MODEL PARAMETERS** | | | |  |  |  |  |  |
|  | N patients analyzed | 86 | 86 | 86 | 86 | 86 | 86 | 84 |
|  | Intercept | -0.39  (-1.19, 0.42) | -0.88  (-1.85, 0.10) | -1.90  (-3.46, -0.34) | -0.98  (-2.05, 0.10) | -1.32  (-2.78, 0.14) | -1.79  (-3.13, -0.45) | -2.71  (-4.83, -0.59) |
|  | Presence of co-infection | 0.07  (-0.54, 0.68) | -0.24  (-1.23, 0.76) | 0.90  (-0.58, 2.38) | 0.02  (-0.61, 0.64) | 0.02  (-0.64, 0.68) | -0.69  (-2.77, 1.39) | -0.08  (-1.23, 1.06) |
|  | *DENV RNA detected in first sample* | 0.02  (-0.49, 0.53) | 0.04  (-0.54, 0.62) | 0.03  (-0.64, 0.70) | -0.01  (-0.61, 0.59) | 0.15  (-0.73, 1.03) | -0.08  (-0.86, 0.70) | 0.34  (-1.32, 1.99) |
|  | Male gender | -0.02  (-0.46, 0.41) | 0.13  (-0.54, 0.79) | 0.03  (-0.50, 0.55) | -0.11  (-0.76, 0.54) | 0.08  (-0.56, 0.72) | 0.11  (-0.65, 0.86) | -0.12  (-1.04, 0.80) |
|  | Presence of chronic disease | 0.04  (-0.51, 0.59) | 0.12  (-0.65, 0.89) | 0.05  (-0.62, 0.72) | -0.18  (-1.14, 0.78) | 0.01  (-0.76, 0.77) | -0.05  (-0.93, 0.82) | -0.05  (-1.20, 1.10) |
|  | Days from onset to first RNA sample | 0.00  (-0.05, 0.06) | 0.00  (-0.06, 0.07) | 0.09  (-0.07, 0.25) | -0.02  (-0.11, 0.08) | -0.03  (-0.16, 0.10) | 0.02  (-0.09, 0.12) | 0.01  (-0.10, 0.12) |
|  | Age (mean-centered) | 0.00  (-0.02, 0.02) | -0.03  (-0.08, 0.02) | -0.01  (-0.04, 0.03) | 0.01  (-0.02, 0.03) | -0.05  (-0.10, 0.01) | -0.02  (-0.07, 0.03) | -0.02  (-0.09, 0.04) |
| **DENV NS1 MODEL PARAMETERS** | | | |  |  |  |  |  |
|  | N patients analyzed | 86 | 86 | 86 | 86 | 86 | 86 | 84 |
|  | Intercept | -0.94  (-2.44, 0.57) | -0.86  (-1.92, 0.21) | -1.95  (-3.47, -0.42) | -0.92  (-2.03, 0.20) | -2.85  (-5.50, -0.20) | -1.73  (-3.07, -0.39) | -14.90  (-∞, ∞) |
|  | Presence of co-infection | 0.14  (-0.64, 0.93) | -0.26  (-1.29, 0.77) | 1.04  (-0.47, 2.54) | 0.01  (-0.61, 0.63) | 0.14  (-0.77, 1.05) | -0.81  (-3.01, 1.39) | -0.01  (-1.14, 1.13) |
|  | *DENV NS1 detected in first sample* | 0.61  (-0.74, 1.96) | 0.14  (-0.65, 0.93) | -0.01  (-0.66, 0.64) | -0.07  (-0.74, 0.59) | 1.55  (-0.92, 4.03) | -0.01  (-0.74, 0.72) | 12.50  (-∞, ∞) |
|  | Male gender | -0.04  (-0.50, 0.42) | 0.04  (-0.47, 0.56) | 0.01  (-0.52, 0.53) | -0.09  (-0.72, 0.53) | 0.15  (-0.65, 0.95) | -0.02  (-0.62, 0.58) | -0.08  (-0.94, 0.78) |
|  | Presence of chronic disease | 0.10  (-0.56, 0.76) | 0.05  (-0.60, 0.71) | 0.05  (-0.62, 0.71) | -0.25  (-1.32, 0.83) | 0.03  (-0.75, 0.81) | -0.10  (-1.05, 0.85) | -0.10  (-1.30, 1.09) |
|  | Days from onset to first NS1 sample | 0.01  (-0.06, 0.09) | 0.01  (-0.06, 0.08) | 0.09  (-0.07, 0.25) | -0.02  (-0.12, 0.08) | -0.01  (-0.12, 0.10) | 0.03  (-0.09, 0.14) | 0.02  (-0.12, 0.16) |
|  | Age (mean-centered) | -0.01  (-0.03, 0.02) | -0.03  (-0.08, 0.02) | -0.01  (-0.03, 0.02) | 0.01  (-0.02, 0.04) | **-0.06**  **(-0.12, -0.01)** | -0.02  (-0.08, 0.03) | -0.02  (-0.08, 0.04) |
| \| ^a^Coefficents on a logit scale. \| \| --- \| \| ^b^Coefficients on a natural logarithmic scale. \| \| Averaged parameter coefficients where confidence intervals exclude zero are written in bold.  Abbreviations: ALT, alanine transaminase; AST, aspartate transaminase; CI, confidence interval; DENV, dengue virus; Hb, hemoglobin; Hcr, hematocrit; NS1, non-structural protein 1 \| \|  \| | | | | | | | | |
