# Supplementary material for: Dengue in Travelers: Kinetics of Viremia and NS1 Antigenemia and Their Associations with Clinical Parameters
Source: PLoS One. 2013 Jun 3;8(6):e65900. doi: 10.1371/journal.pone.0065900 (PMC3670861; doi:10.1371/journal.pone.0065900)
Supplement: Table S3 — Parameter estimates and their 95% confidence intervals from averaged model sets exploring the connection between initial amounts of serum DENV RNA/NS1 antigen and clinical parameters in follow-up. (DOCX) [file pone.0065900.s005.docx]

| **Supplementary Table 3.**  **Parameter estimates with their 95% confidence intervals from averaged model sets exploring the connection between initial amounts of serum DENV RNA / NS1 antigen and clinical parameters in follow-up.** | | | | | | | | | | | | | | |
| --- | --- | --- | --- | --- | --- | --- | --- | --- | --- | --- | --- | --- | --- | --- |
|  | | |  | Coefficients (95% CIs) | | | | | | | | | | |
|  | | |  | **ALT maximum** | | **AST maximum** | | **Leukocyte count nadir** | **Platelet count nadir** | **Hcr**  **maximum** | **Hb maximum** | **Creatinine maximum** | **Hospitali-**  **zation^a^** | **Days hospita-lized^b^** |
| **DENV RNA MODEL PARAMETERS** | | | | | | | |  |  |  |  |  |  |  |
|  | N patients analyzed | | | 78 | | 59 | | 83 | 82 | 82 | 82 | 36 | 86 | 83 |
|  | Intercept | | | 118.00  (40.90, 195.00) | | 107.00  (9.10, 204.00) | | 3.86  (2.66, 5.06) | 128.00 (61.10,  196.00) | 0.411 (0.389, 0.434) | 140.00 (132.00, 148.00) | 159.00  (-4.70, 324.00) | -0.06  (-1.34, 1.23) | 0.79  (0.35, 1.22) |
|  | *DENV RT-PCR inverse ct value in first sample* | | | 2.02  (-3.07, 7.11) | | 5.18  (-2.21, 12.60) | | **-0.13**  **(-0.20,**  **-0.07)** | **-5.70**  **(-8.65,**  **-2.75)** | 0.001  (-0.001, 0.002) | 0.19  (-0.33, 0.70) | -5.28  (-13.70, 3.10) | **0.24**  **(0.08,**  **0.40)** | **0.05**  **(0.02, 0.07)** |
|  | Age (mean-centered) | | | -1.63  (-4.68, 1.41) | | -0.99  (-4.13, 2.16) | | 0.00  (-0.01, 0.02) | -0.47  (-1.80, 0.87) | 0.000 (0.000, 0.001) | 0.06  (-0.16, 0.28) | -0.31  (-2.14, 1.52) | 0.00  (-0.02, 0.03) | 0.00  (-0.01,  0.01) |
|  | Male gender | | | 8.46  (-36.40, 53.30) | | 3.94  (-38.50, 46.40) | | -0.02  (-0.41, 0.37) | -18.00  (-57.20, 21.20) | **0.037 (0.019, 0.055)** | **13.00 (6.32, 19.60)** | 16.20  (-44.00, 76.30) | 0.02  (-0.62, 0.58) | 0.00  (-0.16,  0.17) |
|  | Days from onset to first RNA sample | | | -0.86  (-6.11, 4.39) | | -1.20  (-7.83, 5.44) | | 0.04  (-0.07, 0.15) | 4.97  (-0.45,  10.40) | 0.000  (-0.001, 0.001) | 0.01  (-0.41, 0.42) | -5.69  (-18.60, 7.19) | 0.01  (-0.08, 0.10) | 0.01  (-0.03,  0.04) |
|  | Presence of co-infection | | | 5.77  (-39.30, 50.80) | | 14.20  (-51.90, 80.30) | | 0.54  (-0.62, 1.69) | -8.20  (-41.90, 25.50) | -0.014  (-0.041, 0.012) | -3.50  (-12.20, 5.25) | 27.60  (-55.10, 110.00) | 0.15  (-0.75, 1.04) | 0.12  (-0.27,  0.51) |
|  | Presence of chronic disease | | | 11.30  (-44.20, 66.80) | | 24.60  (-58.90, 108.00) | | 0.21  (-0.58, 1.00) | 3.50  (-21.00, 28.00) | -0.008  (-0.030, 0.014) | -1.55  (-7.87, 4.77) | 79.20  (-23.10, 182.00) | 1.04  (-1.34, 3.43) | 0.07  (-0.23,  0.36) |
| **DENV NS1 MODEL PARAMETERS** | | | | | | | |  |  |  |  |  |  |  |
|  | | N patients analyzed | | | 77 | | 59 | 82 | 81 | 81 | 81 | 35 | 86 | 83 |
|  | | Intercept | | | 61.40  (-21.60, 144.00) | | 47.60  (-43.40, 139.00) | 4.08  (2.69, 5.47) | 125.00 (73.20, 177.00) | 0.386 (0.364, 0.407) | 129.00 (122.00, 137.00) | 173.00  (-4.08, 349.00) | 1.25  (-0.02, 2.52) | 1.28  (0.85, 1.72) |
|  | | *DENV NS1 ratio in first sample* | | | **13.20**  **(2.32, 24.10)** | | **18.60 (7.31, 29.90)** | **-0.30**  **(-0.44,**  **-0.17)** | **-11.60**  **(-17.20,**  **-6.05)** | **0.005 (0.003, 0.008)** | **2.01**  **(1.04, 2.97)** | -12.30  (-30.90, 6.27) | 0.03  (-0.10, 0.15) | 0.01  (-0.03, 0.04) |
|  | | Age (mean-centered) | | | -1.61  (-4.63, 1.41) | | -0.84  (-3.80, 2.11) | 0.00  (-0.02, 0.01) | -0.78  (-2.31, 0.74) | 0.000 (0.000, 0.001) | 0.04  (-0.14,  0.22) | -0.41  (-2.52, 1.70) | 0.00  (-0.02, 0.03) | 0.00  (-0.01, 0.01) |
|  | | Male gender | | | 10.60  (-36.80, 58.00) | | 2.62  (-35.00, 40.30) | 0.06  (-0.37, 0.49) | -3.18  (-23.20, 16.80) | **0.039 (0.022, 0.056)** | **14.20 (8.28,**  **20.00)** | 35.80  (-41.60, 113.00) | 0.15  (-0.91, 0.62) | -0.08  (-0.39, 0.23) |
|  | | Days from onset to first RNA sample | | | -0.41  (-4.67, 3.85) | | -0.80  (-6.20, 4.61) | 0.09  (-0.04, 0.21) | **7.44**  **(3.13, 11.80)** | 0.000  (-0.001, 0.001) | 0.05  (-0.36, 0.46) | -3.89  (-14.80, 7.00) | 0.00  (-0.66, 0.66) | -0.01  (-0.04, 0.03) |
|  | | Presence of co-infection | | | 20.50  (-49.80, 90.70) | | 28.00  (-56.00, 112.00) | 0.20  (-0.61, 1.01) | -27.70  (-79.50, 24.20) | -0.004  (-0.021, 0.013) | -0.68  (-5.23, 3.87) | 22.90  (-58.70, 105.00) | 1.41  (-1.00, 3.81) | 0.06  (-0.25, 0.37) |
|  | | Presence of chronic disease | | | 7.21  (-39.30, 53.70) | | 37.60  (-57.00, 132.00) | 0.11  (-0.49, 0.70) | 1.06  (-18.30, 20.50) | -0.007  (-0.026, 0.013) | -1.14  (-6.31, 4.03) | 37.30  (-48.10, 123.00) | -0.02  (-0.11, 0.07) | 0.16  (-0.28, 0.60) |
| Averaged parameter coefficients where confidence intervals exclude zero are written in bold.  ^a^ Coefficients on a natural logarithmic scale  ^b^ Coefficients on a logit scale  Abbreviations: ALT, alanine transaminase; AST, aspartate transaminase; CI, confidence interval; ct, cycle threshold; DENV, dengue virus; Hb, hemoglobin; Hcr, hematocrit; NS1, non-structural protein 1; RT-PCR, reverse transcription polymerase chain reaction | | | | | | | | | | | | | | |
